# Protein disulfide topology determination through the fusion of mass spectrometric analysis and sequence-based prediction using Dempster-Shafer theory

**DOI:** 10.1186/1471-2105-14-S2-S20

**Published:** 2013-01-21

**Authors:** Rahul Singh, William Murad

**Affiliations:** 1 Department of Computer Science, San Francisco State University, San Francisco, CA 94132, USA

## Abstract

**Background:**

Disulfide bonds constitute one of the most important cross-linkages in proteins and significantly influence protein structure and function. At the state-of-the-art, various methodological frameworks have been proposed for identification of disulfide bonds. These include among others, mass spectrometry-based methods, sequence-based predictive approaches, as well as techniques like crystallography and NMR. Each of these frameworks has its advantages and disadvantages in terms of pre-requisites for applicability, throughput, and accuracy. Furthermore, the results from different methods may concur or conflict in parts.

**Results:**

In this paper, we propose a novel and theoretically rigorous framework for disulfide bond determination based on information fusion from different methods using an extended formulation of Dempster-Shafer theory. A key advantage of our approach is that it can automatically deal with concurring as well as conflicting evidence in a data-driven manner. Using the proposed framework, we have developed a method for disulfide bond determination that combines results from sequence-based prediction and mass spectrometric inference. This method leads to more accurate disulfide bond determination than any of the constituent methods taken individually. Furthermore, experiments indicate that the method improves the accuracy of bond identification as compared to leading extant methods at the state-of-the-art. Finally, the proposed framework is extensible in that results from any number of approaches can be incorporated. Results obtained using this framework can especially be useful in cases where the complexity of the bonding patterns coupled with specificities of the fragmentation pattern or limitations of computational models impair any single method to perform consistently across a diverse set of molecules.

## Background

Disulfide (S-S) bonds constitute one of the main cross-linkages present in proteins and can be broadly characterized to be structural, catalytic, or allosteric [1]. Structural S-S bonds play an important role in the folding and stabilization of proteins and are involved in the formation of structural motifs such as the cysteine knot and CXXC motif. Catalytic S-S bonds mediate thiol-disulfide interchange reactions in substrate proteins and play an important role in the regulation of enzymatic activity [2,3]. Finally, allosteric S-S bonds regulate protein function in non-enzymatic ways by triggering a conformational change when the bond breaks and/or forms. Thus, identification of the S-S bond topology constitutes one of the essential components for understanding and reasoning about both protein structure and function [1].

At the state-of-the-art, several methods can be used for determination of S-S bonds including Edman degradation, NMR, crystallography, and algorithmic methods that are either based on analysis using sequence information (hereafter termed sequence-based methods) or analysis of information from Mass Spectrometry (hereafter called MS-based methods). Recent introductions and reviews of these methods can be found in [1,4,5]. It is important to note that each of the above class of methods has advantages as well as shortcomings. For instance, the use of Edman degradation can be limited due to requirements of ultra-pure samples. Similarly, NMR and crystallography, while highly accurate, require relatively large amounts (10 to 100 mg) of pure protein in a particular solution or crystalline state. Both these methods can also be limited by protein size, and are fundamentally low-throughput.

Amongst approaches that involve algorithmic analysis, sequence-based methods utilize global features, such as the statistical frequency of amino acid residues [6] and cysteine state sequences [7] or local features that encode the characteristics of the sequence environment around the cysteines [8,9]. The process of developing a model for determining the S-S connectivity from such features can be based on: (1) characteristics of nearest neighbour(s). Techniques in this category identify disulfide bonds based on the closest training sample(s) in the feature space [10-12]. From a machine learning perspective, this class of methods constitutes examples of instance-based learning. (2) Supervised learning of the classification function. Methods in this class have employed approaches like neural networks, support vector machines, and logical regression [6,13-15]. (3) Methods based on physics-based modelling. This class of methods has primarily been based on modelling the problem as a graph, where cysteines constitute the vertices and the edges are weighted using some measure that is indicative of physical-chemical interactions, such as contact potential or evolutionary information [16,17]. Determining the disulfide connectivity is then cast as a graph-theoretic optimization problem.

An advantage of sequence-based methods is that once a model has been developed, its application does not require significant data preparation and can be run in high-throughput settings as it only requires the protein sequence information. A critical disadvantage however, lies in the fact that it may not always be possible to obtain an accurate mapping between local or global features and the presence of specific disulfide bonds. For supervised methods, difficulties can also arise if the test samples have high sequence homology with the training set but weaker structural homology.

MS-based methods [18-21] involve a combination of experimental and algorithmic processing and can be applied under conditions of either partial reduction or non-reduction of the protein. The basic idea behind MS-based methods lies in: (1) generating the theoretical spectra in terms of the fragmentation model used by a specific method and (2) matching the theoretical spectra to the experimental spectra obtained from the MS or MS/MS step. While MS-based methods are generally more accurate than sequence-based methods, as shown by the direct comparisons in [22], they too have limitations. For instance, ambiguous results can occur under conditions of partial reduction if the S-S bonds have similar reduction rates. Under non-reduction conditions on the other hand, S-S bonds can be missed for molecules that have multiple S-S bonds or large number of cysteines [21]. Furthermore, the fragmentation model used in the algorithms for interpreting MS-data can also have limitations; commonly used fragmentation models often consider only a small number of ion types to avoid a combinatorial explosion in the number of theoretical fragments that have to be generated and matched [19]. However, other ion types do occur and should ideally, be accounted for. Finally, under certain bond arrangements, the fragmentation process from mass spectrometry may itself lack sufficient information to identify specific bonds. This can happen for example when (1) the precursor ion fragmentation produces different fragments only at the outside boundaries of the intra-disulfide bond, (2) the presence of cross-linked or circular disulfide bonds prevent the fragmentation of precursor ions, or (3) the energy used to fragment complex molecules is not sufficient to break strong intra-chain and inter-chain bonds present in the molecules structure. All the above conditions can cause too few product ions to be generated.

An illustration of the variable success of established S-S bond detection methods as applied to a set of nine eukaryotic Glycosyltransferases is shown in Table 1. While not exhaustive in terms of available methods, the table demonstrates that no single class of method performs accurately in all cases. For instance, the mass spectrometry-based method MassMatrix fails to identify the C24-C145 bond in the molecule C2GnT-I (Swiss-Prot:Q09324). This bond is found by both DISULFIND and DiANNA 1.1, which are sequence-based methods. However, as the reader can see, not all sequence-based methods find this bond. The table also highlights the fact that methods (and underlying models) which work well in some cases don't work equally well in others. For instance, DisLocate, which utilizes protein subcellular localization to determine the S-S bonds, can find only one bond. However, on the SPxx data sets [23], this method has been shown to outperform other sequence-based methods [24].

**Table 1:** Results of S-S bond determination on a set of Glycosyltransferases using MS-based and sequence-based methods.

Swiss-Prot ID	Known S-S bonds	Methods that detected the bond	Methodology
Q92187	*142-292*	MassMatrix	MS-based
	*156-356*	MassMatrix	MS-based
P02754	*82-176*	MassMatrix, DisLocate, DiANNA 1.1	MS-based, Sequence-based
	*122-135*	-	-
Q11130	*68-176*	MassMatrix, PreCys	MS-based, Sequence-based (cysteine separation profile)
	*211-214*	MassMatrix, PreCys	As above
	*318-321*	MassMatrix, PreCys	As above
P08037	*134-176*	MassMatrix	MS-based
	*247-266*	MassMatrix	MS-based
Q09324	*59-413*	-	-
	*100-172*	-	-
	*151-199*	-	-
	*372-381*	-	-
P00698	*24-145*	DISULFIND, DiANNA 1.1	Sequence-based
	*48-133*	MassMatrix, DiANNA 1.1, DISULFIND	MS-based, Sequence-based
P21217	*81-338*	-	-
	*91-341*	-	-

The molecules and their Swiss-Prot ID are: ST8Sia IV [Swiss-Prot:Q92187], Beta-lactoglobulin [Swiss-Prot:P02754], FucT VII [Swiss-Prot:Q11130], C2GnT-I [Swiss-Prot:Q09324], Lysozyme [Swiss-Prot:P00698], FT III [Swiss-Prot:P21217], β1-4GalT [Swiss-Prot:P08037], Aldolase [Swiss-Prot:P00883], and Aspa [Swiss-Prot:Q9R1T5]. The methods investigated include MassMatrix [21], DisLocate [24], DISULFIND [14], PreCys [4], and DiANNA 1.1 [17].

Given the aforementioned context, we propose a novel theoretical framework, as well as a concrete method for S-S bond determination based on aggregation and fusion of evidence from different methods. This framework is based on the Dempster-Shafer theory of evidence combination. As part of our proposed method, we specifically focus on combining evidence from MS-based and sequence-based methods and show that this approach significantly improves upon each of its constituents, in terms of the ability to detect S-S bonds.

### Problem formulation, challenges and requirements for method design

Consider a protein P with n cysteines *C_i_, ... C_n_*. Let the amino acids in the sequence of *P *be numbered from the N-terminus to the C-terminus. Now, let *Ψ *= {*ψ*_1_, *ψ*_2_, ..., *ψ*_m_} be a set of *m *disulfide bond determination techniques, each of which takes as an input some information about *P *and provides as its output the S-S connectivity of *P*. For instance, *ψ*_1 _could be a MS-based method, *ψ*_2 _could be a sequence-based method that uses local descriptors, and so on. Let also each method *ψ_k _*assign some form of a confidence score *σ_k_*(*i, j*) to each cysteine pair (*C_i_*, *C_j_*) that forms a S-S bond. This score would reflect the balance of evidence, as determined by *ψ_k _*based on which (*C_i_*, *C_j_*) was determined to participate in the S-S bond. Without loss of generality, we shall assume this confidence score to be normalized in the interval [0 1]. We shall further denote the S-S connectivity of *P *obtained from method *ψ_k _*as the set *Ɗ_k_*, each element of which is a triplet containing the pairs of bonded cysteine residues along with the corresponding confidence score. That is:(1)

It follows from the discussion in the previous section, that for a protein *P*, the S-S connectivity *Ɗ_1_*, *Ɗ_2_*, ... *Ɗ_k _*obtained using the corresponding methods *ψ*_1_, *ψ*_2_, ..., *ψ*_m_, will in general, not be identical. Our goal is to develop an information fusion-based method *Ɲ *that appropriately combines the connectivity evidence from the methods comprising *Ψ*. Symbolically, we shall denote the combination of evidence from different sources hereafter as:(2)

From an epistemological perspective, information or evidence from different methods can be: (1) *consonant*, that is, the evidence can be represented as a hierarchy, where the elements (S-S bonds) of the smallest set are included in the next larger set and so on. Such a situation can occur, for instance, if information is refined across methods. (2) *Consistent*, here there is one (or more) element(s) that is common to all sets. (3) *Arbitrary*, here some sets may have elements in common but no element is common to all sets. (4) *Disjoint*, there are no common elements for any pair of subsets.

Each of the aforementioned types of evidence has implications for a method that seeks to combine them. In the case of consonant evidence, there is agreement on the smallest set of evidence. However, there can be conflict between the additional evidence present in any given set with respect to its subset. Consistent evidence implies that there is agreement on at least one set of bonds. With arbitrary evidence, there is some agreement amongst some methods but there is no consensus amongst all methods on a specific S-S bond. Finally, in the case of disjoint evidence, each of the methods provides conflicting bond topologies. In combining the evidence from different methods, our method *Ɲ *needs to have the following characteristics:

• The method should not require information about the probability distribution functions of the various sources of information.

• It should be able to quantify the agreement and the conflict amongst the methods.

• It should be able to use methods with potentially high amounts of conflict.

• The results from *Ɲ *should be independent of the order in which the evidence is presented. This would allow us to update the results as new evidence (methods) becomes available.

• The method should be able to incorporate external information (such as input from an expert) on the relative reliability of the S-S bond determination methods.

## Methods

### Dempster-Shafer theory of evidence

Dempster-Shafer Theory (DST) is a mathematical theory of evidence, which in finite discrete spaces, can be treated as a generalization of probability theory. One of the most important features of DST is that it can be used to combine information from multiple sources under conditions of epistemic uncertainty. Specifically, for our problem, DST can provide the theoretical underpinnings for a method which has to deal with information about S-S bonds from different methods that may be consonant, consistent, arbitrary, or disjoint. In the following we briefly introduce the relevant concepts of DST before describing our approach based on its extension. For details of DST, the interested reader is referred to [25,26].

In DST, a frame of discernment *θ *consists of a set of primitive hypotheses or decisions. The frame *θ *must be exhaustive, containing all possible primitive hypotheses (singletons) and have mutually exclusive elements. For example, for a molecule *P *with *n *cysteines, *θ *would be the set of all *n(n-1)/2 *pairs of cysteines, corresponding to all the possible S-S bonds. The basic belief assignment function (also called mass function) *m*, assigns a measure of belief to a decision. Specifically, *m *assigns to each subset of *θ *a number in the range [0 1]. Thus, *m*:2^*θ *^→ [0 1]. Further, *m *also conforms to the properties enumerated in Eq. (3) - Eq.(5)

(3) m(ϕ)=0

(4) $$m\left(A\right)\geq 0,\;\;A\in 2^{\theta }$$

(5) $$\underset{A\in 2^{\theta }}{\sum }m\left(A\right)=1$$

Based on the above constructs and properties, the key distinctions between DST and probability theory can be made: first, probability distribution functions are defined on *θ*, while in DST, the assignment function is defined on the power set: 2^*θ*^. Second, given Eq.(5), the belief not assigned to any subset of 2^*θ *^is assigned to the environment. Third, it is not required that *m*(*A*) ≤ *m*(*B*), if *A *⊂ *B*. Finally, *m*(*A*) and $m\left(Ā\right)$ are not required to be related. That is, knowledge of an event does not require knowledge of its complement. By applying the assignment function, several evidential functions can be constructed. Two commonly used evidential functions in DST are *belief *and *plausibility*. For a given decision *A*, the belief of *A*, denoted as *ß*(*A*), is the measure of how much the information given by a source supports a specific element to be the correct answer. Thus, *ß*(*A*): 2^*θ*^→ [0 1] and *ß*(*A*) is defined as shown in Eq.(6). Correspondingly, the plausibility of *A*, *Ƥ*(*A*) measures how much the information from a source does not contradict a hypothesis. Thus, *Ƥ*(*A*): 2^*θ*^→ [0 1] and its definition is given in Eq. (7). It may be noted that these two measures are non-additive. That is, the sum of all the belief measures (plausibility measures) is not required to be 1. The belief interval of *A *is given by [*ß*(*A*) *Ƥ*(*A*)] and gives the evidential interval range representing the uncertainty associated with the decision *A*. This interval is often interpreted to be the range within which one can believe in the decision *A *without severe errors [27].(6)(7)

### Evidence combination and extension of DST

The classical method for combining independent evidence from different sources is the Dempster rule. Let  be the result of combining the decisions from two methods *ψ*_1 _and *ψ*_2 _using the Dempster rule:(8)

Note that the Dempster rule is commutative, associative, and non-idempotent. It is advantageous to think of the denominator in terms of a normalization factor *X *= *1*/*k*, where *k *equals the denominator in Eq. (8). The quantity log(*X*) is termed the weight of conflict. If there is no conflict between the evidences, the sum of their beliefs equals 1 and the weight of the conflict equals zero. Conversely, if the evidence is disjoint, then the weight of the conflict becomes infinitely large. The Dempster rule can lead to non-intuitive answers in cases where multiple methods have high confidence in disjoint decisions and agree on some decision with low confidence. In such cases, the Dempster rule assigns high belief to the common decision, even though none of the constituent methods had high confidence in it. This effect is illustrated in Example 1.

*Example 1*: Consider the pancreatic trypsin inhibitor protein (PDB:1G6X). The A chain of 1G6X is of length 58 and consists of six cysteines occurring at residue positions 5, 14, 31, 38, 51, and 55, respectively along with three S-S bonds: C5-C55, C14-C38, and C31-C51. For this protein, the set of primitive hypotheses *θ *= {C5-C14, C5-C31, C5-C38, C5-C51, . . . C31-C38, C31-C51, C-38-C51}. Consider now two hypothetical S-S bond determination methods, *M1 *and *M2*, which output the S-S bonds along with a corresponding belief (or confidence) value. For 1G6X, let the two methods provide the following results (we only show the bonds with non-zero belief scores): *M1*(C5-C55) = 0.99, *M1*(C14-C38) = 0.91, *M1*(C31-C51) = 0.86, *M1*(C14-C31) = 0.01, *M2*(C5-C38) = 0.91, *M2*(C51, C55) = 0.89, and *M2*(C14-C31) = 0.01. Consider now the set of results (bonds) from *M1 *and *M2*. As can be seen, the two methods have high belief in elements that are disjoint between them and a low degree of belief in one common element, namely the bond C14-C31. Applying the Dempster rule, we find *m*_12_(C14-C31) = *ß_12_*(C14-C31) = 1.0 and the belief for all the other bonds to be zero. This counter-intuitive result occurs because the denominator in Eq. (8) attributes any mass associated with conflict to the null set. Consequently, the entire probability is assigned to the only common element, even if both the methods have very low belief in this element.

Different methods, which have been proposed for evidence combination, extend DST by using alternatives to Eq. (8), so as to deal with the above conundrum. In this work, we explore the use of three such alternatives. For notational simplicity, we shall describe these rules by considering the combination of two methods (extensions for larger number of methods is straightforward). The first of these alternate rules for evidence combination is called the Yager rule [28]:(9)

In this rule, *A *is the intersection of subsets *B *and *C *of the power set 2^*θ*^. The fundamental difference between the Dempster rule and the Yager rule is that the latter does not normalize out the conflict of evidence. Rather, the belief associated with the conflict is attributed to the universal set and enlarges the degree of ignorance.

The second rule is based on the work of Campos and Cavalcante [27], which we shall abbreviate as the Campos-rule (see Eq. (10)). The idea underlying this rule is to de-rate the beliefs based on the conflict between the evidences and assign the remaining belief to the environment rather than assigning it to the common hypothesis as is done in the Dempster rule.(10)

In Eq. (10), *X *= 1/*k*, where *k *is the denominator of the Dempster rule. Effectively, in the Campos rule, the orthogonal sum of the Dempster rule is divided by (1+log(*X*)), where log(*X*) is the weight of the conflict between the sources.

The final rule we consider is the discount-and-combine rule proposed by Shafer [25]. Hereafter, we shall call this the Shafer rule. The idea of this rule is to apply a discounting function to each specific belief and then combine by averaging as shown in Eq. (11).(11)

In the above equation, 0 ≤ *α_i _*≤ 1 and *i *is an index for a discount function corresponding to the method *ψ_i_*. One of the important result of our research, as we shall show later, is that it is possible to analyze the mass of the precursor ions from tandem mass spectrometry and appropriately discount the belief associated with the corresponding S-S bond in a data-driven manner.

### Constituent methods

The DST-based framework for information fusion can be used with any number and type of S-S bond determination techniques. In this research, we used three independent methods to determine the S-S connectivity of a protein prior to combining the evidence from them. Of the three methods, two were sequence-based and one involved tandem mass spectrometry. The sequence-based methods included a SVM-based predictor that determined S-S bonds by individually considering each cysteine pair, and a cysteine-separation profile (CSP)-based method. For mass spectrometry data, we used a method developed earlier by us called MS2DB+. In the following, we briefly describe each of these three techniques.

The SVM-based pair-wise predictor used by us is based on [29]. In our implementation of this method, two windows, each of size 13, were centered on each pair of cysteines (which may or may not have been disulfide linked) to generate features that captured the respective local environments. Each residue *y *in the window was encoded by a 20-element bit-vector *V_y _= *{*x_1_, x_2_, x_3_, ..., x_19_, x_20_*}, where each bit *x_i _*was set to 1 if the corresponding amino acid was present. Additionally, the distance between pairs of cysteines, denoted as *d_SS _*was used as a feature. Thus each cysteine-pair was represented by a vector containing 521 features (2 cysteines × 13 residues × 20 elements + 1 *d_SS_*). Finally, a SVM classifier was trained to predict S-S bonds based on the above descriptors. For our investigations, an SVM with RBF-kernel was trained using LIBSVM [30]. To construct the training data, a set of manually annotated S-S bonded proteins was extracted from the SWISS-PROT SP43 dataset [31]. Following [16], a filtering procedure was applied to ensure only high quality and experimentally verified S-S bonds were included. The filtering criteria were as follows: (1) only the sequences in the PDB were considered, (2) sequences with S-S bonds annotated as "probable", "potential" or "by similarity" were excluded, and (3) protein sequences with more than five disulfide bonds were also excluded. The filtered dataset contained 439 proteins. The belief score *σ_SVM _*for each predicted S-S bond was calculated as shown in Eq. (12) by following [32]. In Eq. (12), A and B denote the model parameter settings and *f *denotes the estimate of the decision function. The optimal values for A and B were determined by regularized maximum likelihood estimation following [32].

(12) $$\sigma_{SVM}=1/1+e^{Af+B}$$

The second method used by us involved S-S connectivity prediction by matching cysteine separation profiles (CSPs). The idea of CSP was proposed in [12] and is based on the observation that proteins with similar disulfide bonding patterns share similar folds. Consequently, the separation between oxidized cysteine residues (CSP) can be used for determining disulfide connectivity. Given a protein *P *with 2*n *cysteine residues *C_1_, C_2_, ... C_2n_*, its cysteine separation profile is defined as:

(13) $$\text{CSP}\left(P\right)=\left(C_{2}\text{-}C_{1},C_{3}\text{-}C_{2},\ldots C_{2n}\text{-}C_{2n\text{-}1}\right)$$

Further, the divergence *D *between two *CSPs *is defined in Eq. (14), where $s_{i}^{X}$ and $s_{i}^{Y}$ are the *i*th separations for *CSPs *of two different proteins *X *and *Y*. The S-S connectivity of a protein *P *is inferred by comparing the CSP of *P *against a database from proteins with manually annotated disulfide bonds (in our work a database of 439 proteins, filtered from the SwissProt SP43 dataset, was used). Specifically, the disulfide connectivity of *P *is predicted to be same as that of a database protein having the most similar cysteine separation profile. In spite of its conceptual simplicity, the above idea has been found to perform well in practice [12]. In our adaptation of this method, the belief score for a bond was defined to be inversely proportional to the divergence (Eq. (15)).

(14) $$D=\underset{i}{\sum }|s_{i}^{X}-s_{i}^{Y}|$$

(15) $$\sigma_{CSP}={\left(1+\log_{10}\left(1+\frac{D}{10}\right)\right)}^{-2}$$

As the third method, we used a tandem mass spectrometry-based approach called MS2DB+, which was proposed by us in [19]. This method employs an expanded fragmentation model that considers multiple ion-types (a, a_o_, a*, b, b^o^, b*, c, *x*, y, y^o^, y*, and *z*). To manage the exponential growth of the search space due to the consideration of so many ion types (note that most MS/MS based methods tend to account for *b/y *ions only), MS2DB+ utilizes an efficient approximation algorithm for matching the experimental and theoretical spectra. Specifically, after filtering the theoretical S-S bonds by using the precursor ion mass as a threshold, the method identifies from among the remaining disulfide-bonded peptide fragments, those with mass close to the given experimental spectra. This problem can be thought of as the subset-sum problem, where the goal is to determine the pair (*S*, *t*), where *S *corresponds to the set of disulfide-bonded peptide fragments and *t *corresponds to the targeted mass value from the experimental spectra. Next, a near optimal solution is found using an approximation algorithm which trims as many elements as possible from the search space based on a data-derived trimming parameter *ε*. For the search space *DMS *consisting of the set of mass values corresponding to every possible disulfide bonded peptide structure for the protein, the trimming process removes as many elements as possible to create the trimmed set DMS*, such that for every element *DMS_i _*removed from *DMS*, there remains an element $DMS_{i}^{*}\in DMS^{*}$ which is "close" in terms of its mass to the deleted element *DMS_i_*:

(16) $$\left(DMS_{i}/1+\varepsilon \right)\leq DMS_{i}^{*}\leq DMS_{i}$$

Each match found is further validated in a confirmatory phase to eliminate any correspondences due to chance and obtain a "local" (bond-level) view of the possible disulfide connectivity. This local information is next integrated to obtain a globally consistent view. Specifically, the location of the putative disulfide bonds is modelled by edges in an undirected graph *G *(*V*, *E*), where the set of vertices *V *corresponds to the set of cysteines. To each edge, a match score representing the combined importance of each single peak match within the two spectra is assigned and each specific peak match is weighted according to its intensity. The match score is given by:

(17) $$V_{S}=\left(\overset{n}{\underset{i=1}{\sum }}\left(VM_{i}\times I_{N}\right)/\overset{n}{\underset{i=1}{\sum }}\left(TMS_{i}\times I_{N}\right)\right)\times 100$$

In Eq. (17), the numerator corresponds to the sum of each validation match for a S-S bond multiplied by the matched MS/MS fragment normalized intensity value (*I_N_*). Here, *VM_i _*is a binary value which is set to 1 if a confirmatory match is found for fragment *i*. Similarly, the denominator contains the sum of each experimental MS/MS fragment ion from *TMS *multiplied by *I_N_*. Here, *TMS_i _*is a binary variable which indicates the presence of a fragment *i *in the MS/MS spectrum. Finally, the globally consistent bond topology is found by solving the maximum weight matching problem for the graph *G*. In addition to the empirical match score of Eq. (17), a probability based scoring model proposed in [21] is also implemented. This model provides two scores called *pp *and *pp_2_*. The *pp *score helps to evaluate whether the number of confirmed matches could be random. The *pp_2 _*score evaluates whether the total abundance (intensity) of the confirmed matches could be random. We use the *pp *score to represent the belief score for a bond as shown in Eq. (18).

(18) $$pp=-\log\left(\sum_{x=n_{match}}^{n}\frac{n!}{x!\left(n-x\right)!}p_{2}^{x}{\left(1-p_{2}\right)}^{n-x}\right)$$

(19) $$p_{2}=\frac{2m\times VM_{TH}}{r}$$

In Eq. (18), *n_match _*represents each confirmatory match found between a product ion and a theoretical fragment ion and *p_2 _*denotes the probability that a product ion randomly matches any of the fragment ions in the theoretical spectrum. Eq. (19) is used for calculating the value of *p_2_*. In it, *m *denotes the product ion mass value; *VM_TH _*indicates the confirmatory match threshold, and *r *denotes the spectrum (mass) detection range.

### Assignment of belief to subsets of putative bonds

If a method outputs |*θ*| primitive hypotheses, then its power set will contain *2^θ ^*subsets. For a given method, let *σ_i _*be the confidence score calculated for a primitive disulfide bond in a subset *j *and let *m(j) *be the belief assignment function for a subset *j *of the power set. Our goal is to design the function *m *which will assign a measure of belief to a set of bonds based on the belief scores for each individual bond in this set. Towards this, we note that the elements in the power set 2^*θ *^can be grouped into two categories: *inconsistent *and *consistent*. The former category contains subsets of the power set containing S-S bonds that share a cysteine. Consequently for an inconsistent subset *j*, the *m(j) *value should be assigned to zero. Alternatively, if a subset contains a single S-S bond, the definition of the assignment function is trivial and equals the belief associated with that bond. The challenge arises when the belief values *m(j) *need to be calculated for the consistent subsets of the power set containing more than one disulfide bond.

Our design of the function *m *is guided by the postulate that the extreme belief score values, that is, zero or very high belief scores, should contribute to the final *m(j) *score in higher significant manners than close-to-average scores. The rationale behind this postulate is that most S-S bond determination methods assign extreme belief scores on a putative bond *only *when the corresponding outcome has a high degree of certainty based on their underlying model. Of course, the underlying model could itself be incorrect. In such situations, the inconsistency in the results is dealt with at the level of evidence combination as described earlier in the section *Evidence combination and extensions of DST*.

The belief assignment function *m*(*j*) proposed by us is shown in Eq. (20). Here, *j *denotes a subset of the power set 2^*θ *^and *k *denotes the different probability scores assigned to the subset *j*. The value of *γ *is defined as in Eq. (21).

(20) $$m\left(j\right)=\max\left\{\left(\overset{k}{\underset{i=1}{\sum }}\left(2^{\sigma i+\gamma }-1\right)/k\right),\;\;0\right\}$$

(21) $$\gamma =\left\{\begin{array}{c} -1\times \overset{k}{\underset{i=1}{\sum }}\sigma_{i}/2k,\;if\;\sigma_{i}=0\\ \overset{k}{\underset{i=1}{\sum }}\sigma_{i}/4k,\;\;if\;\;\sigma_{i}>0 \end{array}\;\right\}$$

In Eq. (20), note that the selection of the maximum confidence belief value between *m*(*j*) and zero prevents negative belief assignments. The addition of factor γ in the equation appropriately accounts for the zero/high confidence scores. Finally, both low scores and close-to-average scores are only slightly modified.

### Discounting uncertain evidence

In general, the performance of different S-S bond determination techniques tends to differ. For example, MS-based methods tend to identify S-S bonds more accurately as compared to sequence-based methods, unless the fragmentation process does not provide sufficient information. Within sequence-based approaches, such distinctions can also be found. In our investigations, for instance, the CSP-based approach correctly determined only 8 out of 17 disulfide bonds (47% sensitivity). Thus, the ability to appropriately discount uncertain evidence from methods can help in improving the accuracy of results. Furthermore, by using the Shafer-rule (Eq. (11)) we can directly utilize the results of the discounting process during evidence combination. In this section, we present a data-driven approach for discounting evidence for MS-based methods. We also discuss empirical strategies for discounting evidence for the SVM- and CSP-based methods used by us.

The accuracy of S-S bond determination using MS-based methods suffers if the precursor ions are large (typically when the mass of the precursor ion is greater than 4000 Da), due to the difficulty in fragmenting large ions. We use this observation to design a function for discounting the evidence from MS-based methods in a data driven manner. In our approach, the weight of each belief score is initially set to the maximum value of 1. That is, there is no discounting to begin with. Subsequently, this weight may be decreased using two data-driven parameters *α_mass _*and *α_pp_*. The value of *α_mass _*depends on the size of the precursor ion matched while that of *α_pp _*depends on the *pp_2 _*value, which is defined as [21]:

(22) $$pp_{2}=-\log\left(\overset{\infty }{\underset{l_{match}}{\int }}\frac{e^{\frac{-{\left(x-\mu_{y}\right)}^{2}}{2\sigma_{y}^{2}}}}{\sqrt{2\pi \sigma_{y}}}dx\right)$$

**Figure 1:**
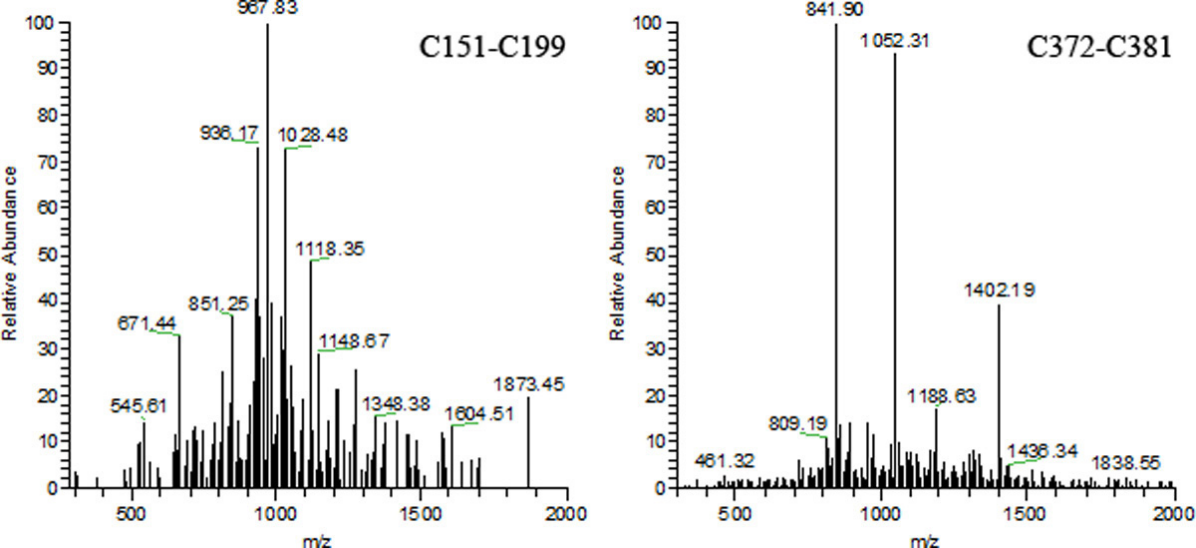
**Tandem mass spectra for protein C2GnT-I**. (Left) Confirmatory spectrum for disulfide bond C151-C199 for protein C2GnT-I (chymotryptic digested). (Right) Confirmatory spectrum for disulfide bond C372-C381 determined for protein C2GnT-I (tryptic digested).

In Eq. (22), *I_match _*represents the abundance (intensity) of an experimental product ion matched to a theoretical fragment ion, *σ_y _*represents the variance for the distribution of the abundance of *i*_th _product ion, and *μ_y _*represents the mean for the distribution of the abundance of *i*_th _product ion. If the precursor ion mass exceeds a threshold *T_mass_*, then the weight is decreased by *α_mass_*. Similarly, if the *pp2 *value assigned to the belief score is lower than a threshold *T_pp_*, then the weight is further decreased by *α_pp_*. In all our experiments, we used the values: *T_mass _*= 4000 Da, *T_pp _*= 50, *α_mass _*= 0.1, *α_pp _*= 0.2. As an example, consider Figure 1 which contains two spectra for S-S bonds in the protein C2GnT-I. The spectrum on the left corresponds to the confirmatory match for the bond between cysteines C151-C199 while the spectrum on the right of the figure corresponds to the bond between cysteines C372-C381. While the precursor ion mass for the former bond is close to 3200 Da, the precursor ion mass size for the later bond exceeds 4200 Da. Consequently, the belief assigned to the bond C372-C381 is decreased by *α_mass _*(= 0.1). Further, in the spectrum associated with C372-C381, there aren't many peaks with intensity higher than 10% of the intensity range (a threshold used by MS2DB+), but all the peaks which exceed the threshold have very high abundance values. Because of this, the total abundance of the confirmed matches (and the *pp_2 _*score) is high. In the spectrum associated with C151-C199, a number of peaks have intensity between 10% and 20% of the intensity range. This leads to a *pp2 *score that is less than *T_pp_*. Consequently, the belief assigned to the bond C151-C199 is decremented by *α_pp_*(= 0.2).

For the evidence from the SVM- and CSP-based methods, the following empirical discounting rules are used: for the SVM-based method, the weight is determined based on the belief score. It is initially set to the same value of the belief score (weight = belief). If the belief score is lower than 0.9 but higher than 0.5, the weight is divided by 1.5. If the belief score is lower than 0.5, the weight is divided by 3. Finally, for the CSP-based method, the weight is calculated based on the divergence score of the matched CSPs and the number of CSP matches containing the lowest divergence score. It is initially set to 1. If the divergence *D *is greater than 10, a decrease of *D*/100 is applied. A penalty of 0.1 is also applied if there are less than two CSP matches with the lowest divergence score determined.

## Results

We investigated the performance of the proposed method (involving the fusion of evidence from MS-based and sequence-based approaches) using a case study and five sets of experiments. In these experiments, we used data from seven eukaryotic glycosyltransferases. These molecules and their Swiss-Prot ID were: ST8SiaIV [Swiss-Prot:Q92187], Beta-lactoglobulin (Beta-LG) [Swiss-Prot:P02754], FucT VII [Swiss-Prot:Q11130], β1-4GalT [Swiss-Prot:P08037], C2GnT-I [Swiss-Prot:Q09324], Lysozyme [Swiss-Prot:P00698], and FT III [Swiss-Prot:P21217]. The MS/MS data was generated following the protocols described in [33,34]. The performance of the method was quantitatively characterized using the following established metrics: *Accuracy *(see Eq. (23)), *Sensitivity *(see Eq. (24)), *Specificity *(see Eq. (25)), and *Matthew's correlation coefficient*, abbreviated hereafter as *MCC *(see Eq. (26)). If the set of disulfide bonds are denoted by *P *and the set of cysteines not forming disulfide bonds by *N*, then true positive (*TP*) predictions occur when disulfide bonds that exist are correctly predicted. False negative (*FN*) predictions occur when bonds that exist are not predicted as such. Similarly, a true negative (*TN*) prediction correctly identifies cysteine pairs that do not form a bond. Finally, a false positive (*FP*) prediction, incorrectly assigns a disulfide link to a pair of cysteines, which are not actually bonded.

(23) $$Accuracy:\;Q_{2}=\left(TP+TN\right)/\left(P+N\right)$$

(24) $$Sensitivity:\;Q_{c}=TP/P$$

(25) $$Specificity:\;Q_{nc}=TN/N$$

(26) $$\text{MCC}:\;c=TP\times TN-FP\times FN/\sqrt{\left(TP+FN\right)\times \left(TP+FP\right)\times \left(TN+FP\right)\times \left(TN+FN\right)}$$

### Performance of the individual methods

The performance of each of the three methods taken separately on this data set is shown in Table 2 and Table 3. These results constitute the baseline. As can be seen from Table 2, the MS-based method MS2DB+ outperformed the two other sequence-based methods. However, the sequence-based methods were able to correctly identify certain S-S bonds, such as C122-C135 (Beta-LG) and C100-C172 (C2GnT-I), which were missed by the MS-based method. Furthermore, for a number of bonds, the methods provided consistent evidence.

**Table 2:** Baseline: the S-S bonds determined by MS2DB+, SVM, and CSP.

Proteins	Bonds determined by different methods			
	
	Known Linkages	MS2DB+	SVM	CSP
ST8Sia IV	**142-292**	142-292	142-292	142-292
	**156-356**	156-356	156-356	156-356

Beta-LG	**82-176**	82-176	-	-
	**122-135**	-	122-135	122-135
		-	*137-176*	*137-176*

FucT VII	**68-76**	68-76	68-76	68-76
	**211-214**	211-214	211-214	211-214
	**318-321**	318-321	318-321	318-321

Β1,4-GalT	**134-176**	134-176	-	134-176
	**247-266**	247-266	-	247-266
		-	*134-247*	-

C2GnT-I	**59-413**	59-413	-	-
	**372-381**	372-381	372-381	-
	**100-172**	-	100-172	-
	**151-199**	151-199	-	-
		-	*199-235*	-
		-	-	*151-217*
		-	-	*172-199*
		-	-	*59-100*

Lysozyme	**24-145**	24-145	24-145	-
	**48-133**	48-133	-	-
		-	-	*82-98*
		-	-	*94-112*

FT III	**81-338**	81-338	-	-
	**91-341**	-	-	-
		-	*81-91*	*81-91*
		-	*338-341*	*338-341*

False positives are indicated in italics.

**Table 3:** The overall performance of MS2DB+, SVM, and CSP.

Methods	*Q_c_*	*Q_nc_*	*Q_2_*	c
**MS2DB+**	0.821	1.000	0.982	0.891
**SVM**	0.571	0.977	0.964	0.581
**CSP**	0.500	0.969	0.959	0.459

### Case study to illustrate the framework

We illustrate the working of the proposed method by analyzing the molecule β1,4GalT. The true S-S connectivity pattern for this molecule is known to be {(134-176), (247-266)}. Table 4 presents all the disulfide bond determination steps of the proposed method in order. From top to bottom, the framework starts by determining the initial connectivity found by each constituent method along with their corresponding belief scores. The reader may note that both the CSP-based method and the MS-based method found the correct topology. However, the belief scores assigned by the CSP-based method were in the middle-to-low range. By contrast, the SVM method had high confidence scores assigned to a bond that turned out to be incorrect.

**Table 4:** Illustration of the key steps in the proposed method

Correct S-S topology: C134-176, C247-C266		
Initial Connectivity {pairs(bond, belief)}		
MS/MS	SVM	CSP
*(134-176, 0.80)*	*(134-247, 0.96)*	*(134-176, 021)*
*(247-266, 0.81)*		*(247-266, 0.21)*
Primitive hypothesis		
*{(134-176), (247-266), (134-247)}*		
Power Set		
*{(134-176), (247-266), (134-247), {(134-176),(247-266)}}*		
Power set scoring (per method)		
MS/MS	SVM	CSP
*(134-176, 0.34)*	*(134-176, 0.00)*	*(134-176, 0.20)*
*(247-266, 0.33)*	*(247-266, 0.00)*	*(247-266, 0.20)*
*(134-247, 0.00)*	*(134-247, 1.00)*	*(134-247, 0.00)*
*{(134-176,247-266), 0.33}*	*{(134-176,247-266), 0.00}*	*{(134-176,247-266), 0.20}*
Global S-S connectivity (per combination rule)		
Dempster rule	Campos rule	Shafer rule
*(134-176, 0.47)*	*(134-176, 0.15)*	*(134-176, 0.35)*
*(247-266, 0.47)*	*(247-266, 0.15)*	*(247-266, 0.36)*
*Yager rule: no S-S bonds were found (belief assignments were lower than 0.01)		

The primitive hypotheses for each method are defined based on the initial linkages. The power set of these hypotheses is then generated. The next step involves the computation of the belief assignments, which is done separately for each method and then the values are normalized. The results for each method are presented as a pair (*bonds, belief*) in Table 4. The different combination rules are applied once all the belief scores have been calculated for the bonds constituting the power set (for each of the three constituent methods). An important effect of evidence combination can be seen in this case study: the DST-based approach (utilizing three combination strategies: *Ɲ^Dempster ^,Ɲ^Campos ^, and Ɲ^Shafer^*) was able to successfully combine conflicting evidence from multiple sources to find the right S-S bonding topology. Most interestingly, one of the constituent methods (SVM) suggested an incorrect bond with high confidence. Yet, the proposed approach was able to recover without any a priori knowledge about this method or the data.

### Combination of the different methods using the Shafer rule

In Table 5, we present the S-S bonds along with their corresponding belief scores found for the molecules in the data set. These results were obtained by combining the MS-based MS2DB+ method with the two sequence-based methods using the Shafer rule. The numeric characterization of the performance of the combination of these three methods using the Shafer rule is presented in Table 6. These metrics demonstrate that the combined approach outperformed each of the individual methods and that only one bond (C91-C341) for the protein FT III, was not detected. Further, the results showed an improvement in the overall sensitivity scores when all three methods were combined as compared to the baselines for each individual method.

**Table 5:** Known and determined S-S bonds for all molecules by combining MS2DB+, SVM, and CSP using the Shafer rule.

Protein	Known Bonds	Bonds Found	Belief
ST8Sia IV	**142-292, 156-356**	**142-292, 156-356**	0.66, 0.68
Beta-LG	**82-176, 122-135**	**82-176, 122-135**	0.49, 0.36
FucT VII	**68-76, 211-214**, **318-321**	**68-76, 211-214**, **318-321**	0.43, 0.26, 0.54
Β1,4-GalT	**134-176, 247-266**	**134-176, 247-266**	0.35, 0.36
C2GnT-I	**59-413, 100-172, 151-199, 372-381**	**59-413, 100-172, 151-199, 372-381**	0.07, 0.08, 0.26,0.06
Lysozyme	**24-145, 48-133**	**24-145, 48-133**, 82-98, 94-112	0.31, 0.13, 0.10, 0.10
FT III	**81-338, 91-341**	**81-338**	0.67

The true bonds are shown in bold.

**Table 6:** Quantitative characterization of the combination of MS-based and sequence-based methods (MS2DB+, SVM, and CSP) using the Shafer rule.

Protein	*Q_c_*	*Q_nc_*	*Q_2_*	c
ST8Sia IV	1.00	1.00	1.00	1.00
Beta-LG	1.00	1.00	1.00	1.00
FucT VII	1.00	1.00	1.00	1.00
Β1,4-GalT	1.00	1.00	1.00	1.00
C2GnT-I	1.00	1.00	1.00	1.00
Lysozyme	1.00	0.94	0.94	0.69
FT III	0.50	1.00	0.95	0.69
**Average**	**0.929**	**0.992**	**0.985**	**0.911**

This was due to the correct identification of two disulfide bonds which could not be detected by MS2DB+. Consequently, the accuracy (*Q_2_*) of the proposed method exceeded that of MS2DB+. At the same time, there was a small decrement in the specificity (*Q_nc_*) due to the fact that two false positive bonds were found (for the protein Lysozyme). It may be noted that the loss in specificity was slightly greater for the combination of MS2DB+, SVM and CSP, as compared to the combination of MS2DB+ and SVM alone owing to the fact that the CSP-based method introduced a larger number of false positives when compared to other methods (see Table 2).

### Analysis of all the combination rules

In this experiment, we analyzed the performance of the four different combination rules when applied to the results obtained from all the three methods considered by us: MS2DB+, SVM-based bond determination, and CSP-based bond determination. The results are presented in Table 7. For purposes of comparison, the performance indices of MS2DB+ on this data set are also provided in this table. Overall, the results obtained when using the Shafer rule outperformed the results obtained using any of the other three rules as well as those from MS2DB+. Considering the sensitivity measure (number of bonds determined correctly) as being the most important performance measurement, we note that the Dempster rule as well as the Campos rule also outperformed MS2DB+. The Yager rule had the lowest sensitivity. However, it had better specificity as compared to the Dempster rule and the Campos rule, since it suppressed two false positive bonds (C81-C91 and C338-C341) for the molecule FT III. This result indicates that for a general dataset, no single combination rule can be assumed to always give the best result when considering all the performance metrics (*Q_2_, Q_c_, Q_nc_*, and *c*) and that the results from each of the rules should be analyzed in conjunction with the belief scores. These results also demonstrate that each method contributes to the improvement in performance; while MS2DB+ was responsible for finding most of the S-S bonds, the other two methods (SVM and CSP) were also important for finding the bonds missed by MS2DB+ (C122-C135 for Beta-LG and C100-C172 for C2GnT-I). The bond C91-C341 for the molecule FT III was not found by any of the methods. This bond was also missed by all other S-S bond determination methods that were tested by us (MassMatrix, DISULFIND, PreCys, DiANNA, and DISLOCATE).

**Table 7:** Numeric characterization of the four rules

Methods	*Q_c_*	*Q_nc_*	*Q_2_*	c
**MS2DB+**	0.821	1.000	0.982	0.891
**Dempster rule**	0.857	0.992	0.978	0.772
**Yager rule**	0.714	0.992	0.965	0.589
**Campos rule**	0.857	0.977	0.965	0.797
**Shafer rule**	0.929	0.992	0.985	0.911

### Analysis of the combination of MassMatrix with the sequence-based methods SVM and CSP

One of the advantages of the proposed framework is that it can be applied to any set of S-S bond determination methods. In this experiment, we illustrate and analyze this aspect by combining the results from MassMatrix, with the SVM-based method and the CSP-based method. On our data set, MassMatrix found 9 of the 17 disulfide bonds, while the SVM method found 9 disulfide bonds, and the CSP method found 8 bonds.

Given the performance of MassMatrix, a significant improvement in sensitivity was achieved after combining the results from these three methods using all four combination rules. Specifically, use of the Dempster, Campos, and Shafer rules led to correct identification of 12 of the 17 known bonds by combining the results from the three methods. When compared to MassMatrix, the following bonds were successfully added: C122-C135 (Beta-LG), C372-C381 (C2GnT-I), and C24-145 (Lysozyme). By contrast, the Yager rule found 10 of 17 disulfide bonds, missing the linkages C134-C176 and C247-C266 for the molecule β1,4GalT (these bonds had belief scores lower than *0.01 *and were therefore discarded). These results are presented in Table 8. It is also important to note that including evidence from the sequence-based methods caused five false positive bonds to be reported. These included three false positive bonds (C59-C100, C151-C217, C199-C235) for the molecule C2GnT-I and two false positive bonds (C82-C98, C94-112) for the molecule Lysozyme. This number is higher than what was observed for the combination of the sequence-based methods with MS2DB+, where only two false positive bonds (for Lysozyme) were found in the final results. In the following, we analyze the reasons which led to the greater number of false positives when results from MassMatrix were combined with the sequence-based methods as compared to when results from MS2DB+ were combined.

**Table 8:** Results from combining MassMatrix with the sequence-based methods.

Swiss-Prot ID	Known Bonds	MassMatrix	Dempster, Campos, and Shafer rules	Yager rule
Q92187	**142-192**	142-292	142-292	142-292
	**156-356**	156-356	156-356	156-356
P02754	**82-176**	82-176	82-176	82-176
	**122-135**	-	122-135	122-135
Q11130	**68-176**	68-76	68-76	68-76
	**211-214**	211-214	211-214	211-214
	**318-321**	318-321	318-321	318-321
P08037	**134-176**	134-176	134-176	-
	**247-266**	247-266	247-266	-
Q09324	**59-413**	-	-	-
	**100-172**	-	-	-
	**151-199**	-	-	-
	**372-381**	-	372-381	372-381
P00698	**24-145**	-	24-145	24-145
	**48-133**	48-133	48-133	48-133

When the sequence-based methods were combined with MS2DB+, the three false positive bonds (C59-C100, C151-C217, and C199-C235) for the molecule C2GnT-I were suppressed by true disulfide bonds found by MS2DB+ (C59-C413 and C151-C199). While the match scores for the three false positive bonds equaled 0.55, with MS2DB+, the two true positive bonds C59-C413 and C151-C199, had match scores of 0.70 and 0.80 respectively. As it can be seen, the true bonds shared some of the same cysteines as the false positive bonds. Thus, the true positive bonds outscored the false positive bonds and eliminated them. Since MassMatrix did not find any true positive bonds for the molecule C2GnT-I, the false positives found by the CSP method could not be eliminated. However, it is important to note that the final belief scores for these false bonds, after undergoing the information-fusion step were *lower *than the original belief scores (*σ_CSP_*) found by the CSP method. Specifically, using the Shafer rule, the belief scores for the bonds C59-C100, C151-C217, and C199-C235 were respectively 0.18, 0.08, and 0.03, while the original belief scores for all these bonds equalled 0.55. Using the Dempster rule, the belief scores were 0.06, 0.28, and 0.09, respectively; while for the Yager rule, the belief scores were 0.11, 0.52, and 0.16. Finally, when applying the Campos rule, the belief scores for these bonds were 0.08, 0.39, and 0.12 respectively. The significantly lower belief scores after information fusion may be used as an indication of the lack of certainty in these specific bond assignments.

## Conclusions

In this paper we have presented a novel rigorously grounded framework for S-S bond determination based on combining results from conceptually different methods using extended Dempster-Shafer theory. The proposed approach makes no assumptions about its constituent methods and can deal with significant conflict of evidence in a rigorous manner. Based on this framework, we have proposed a method in which evidence from MS-based methods can be combined with evidence from sequence-based approaches. Experimental results conducted on molecules with varying disulfide-bond topologies indicate that the results obtained with this method improve the rate of bond identification when compared to some of the leading MS-based and sequence-based methods at the state-of-the-art. Additionally, the proposed framework can also be used for exploratory analysis of the possible disulfide connectivity of a molecule by analysing it using cardinally different methods. A web-based implementation of a method based on the proposed framework, called MS2DB++, is publicly available at http://haddock2.sfsu.edu/~ms2db/ms2db++/.

## Competing interests

The authors declare that they have no competing interests.

## Authors' contributions

The method was designed by RS and implemented by WM. Computational studies and experiments were carried out by WM and RS. The paper was written by RS.

## Declarations

Funding for this research and publication was provided by the National Science Foundation grant IIS-0644418 (CAREER).

This article has been published as part of *BMC Bioinformatics *Volume 14 Supplement 2, 2013: Selected articles from the Eleventh Asia Pacific Bioinformatics Conference (APBC 2013): Bioinformatics. The full contents of the supplement are available online at http://www.biomedcentral.com/bmcbioinformatics/supplements/14/S2.
